# The Orexin System in the Enteric Nervous System of the Bottlenose Dolphin (*Tursiops truncatus*)

**DOI:** 10.1371/journal.pone.0105009

**Published:** 2014-08-21

**Authors:** Claudia Gatta, Finizia Russo, Maria Grazia Russolillo, Ettore Varricchio, Marina Paolucci, Luciana Castaldo, Carla Lucini, Paolo de Girolamo, Bruno Cozzi, Lucianna Maruccio

**Affiliations:** 1 Department of Veterinary Medicine and Animal Productions, University of Napoli “Federico II”, Napoli (NA), Italy; 2 Department of Sciences and Technologies, University of Sannio, Benevento (BN), Italy; 3 Department of Comparative Biomedicine and Food Science, University of Padova, Legnaro (PD), Italy; Laboratorio de Neurociencias Moleculares e Integrativas. Escuela de Medicina, División Ciencias de la Salud. Universidad Anáhuac Mayab. Mérida, Yucatán. México, Mexico

## Abstract

This study provides a general approach to the presence and possible role of orexins and their receptors in the gut (three gastric chambers and intestine) of confined environment bottlenose dolphin. The expression of prepro-orexin, orexin A and B and orexin 1 and 2 receptors were investigated by single immunostaining and western blot analysis. The co-localization of vasoactive intestinal peptide and orexin 1 receptor in the enteric nervous system was examined by double immunostaining. Also, orexin A concentration were measured in plasma samples to assess the possible diurnal variation of the plasma level of peptide in this species. Our results showed that the orexin system is widely distributed in bottlenose dolphin enteric nervous system of the all gastrointestinal tract examined. They are very peculiar and partially differs from that of terrestrial mammals. Orexin peptides and prepro-orexin were expressed in the main stomach, pyloric stomach and proximal intestine; while orexin receptors were expressed in the all examined tracts, with the exception of main stomach where found no evidence of orexin 2 receptor. Co-localization of vasoactive intestinal peptide and orexin 1 receptor were more evident in the pyloric stomach and proximal intestine. These data could suggest a possible role of orexin system on the contractility of bottlenose dolphin gastrointestinal districts. Finally, in agreement with several reports, bottlenose dolphin orexin A plasma level was higher in the morning during fasting. Our results emphasize some common features between bottlenose dolphin and terrestrial mammals. Certainly, further functional investigations may help to better explain the role of the orexin system in the energy balance of bottlenose dolphin and the complex interaction between feeding and digestive physiology.

## Introduction

Dolphins, like all toothed whales (odontocetes) mainly feed on fish and squid. Since chewing is an impossible act to perform underwater, dolphins grab their prey and ingest it whole, leaving mechanical digestion to the first muscular chamber of their polygastric stomach complex [1]. Frequent feeding is requested by the intense energetic costs of continuous swimming and the whole structure of the gastroenteric apparatus reflects these needs (i.e. cetaceans have no gallbladder). However the whole organization of the gustatory sense is poorly understood: odontocetes have no sense of smell and the tongue of adult dolphins possesses few or no taste buds [2]. Muscular species of fish and cephalopods living in the proximity of the continental shelf are among the favorite prey of bottlenose dolphins [3]; captive bottlenose dolphins are known to prefer capelin (*Mallotus villosus*) and other fish with a relatively high content of water, especially during the warm season [4]. But it is unclear how gustatory discrimination between different varieties of fish occurs. Feeding preferences (when the abundance of food allows them) may therefore rely on mechanisms other than stimulation of receptors in the mouth.

Orexin A (OXA) and B (OXB), initially identified in the “feeding center” of the rat lateral hypothalamus [5], [6], are respectively 33- and 28- amino acids peptides that originate from a single precursor produced by the prepro-orexin (PPO) gene [5], [6]. Intracerebroventricular (ICV) administration of OXA or OXB is able to increase food intake [5], [7]–[9], hence the name “Orexin” that derives from the Greek word “orexis” [ő*ρεξις*] which means appetite, desire.

OXA is more powerful than OXB, and the actions of both are mediated through binding to the closely related orexin 1 (OX1R) and orexin 2 (OX2R) receptors that belong to the family of G-protein-coupled receptors [5]. While OX1R is highly selective for OXA, OX2R binds both orexins with similar affinity [5].

The orexinergic system is widely distributed throughout the Central Nervous System (CNS) [10]–[13] and in peripheral organs and body systems, including heart [14], adipose tissue [15], kidney [16], endocrine glands [14], [17]–[19], urogenital [20], [21] and gastrointestinal tracts [22], [23]. In addition to a key role in food intake, orexins are also involved in the control of several biological functions such as arterial blood pressure and heart rate [24], sleep/wake cycle [25], water assumption [26], plasma corticosteroid levels [27], testosterone production [28], and endocrine and exocrine pancreatic secretions [29]–[31]. A series of recent studies [32]–[35] proposed a role of the orexin system in the central and peripheral control of gastrointestinal secretion and motility.

In a previous publication on the neuroendocrine regulation of the digestive post-diaphragmatic functions in sea mammals, we reported the distribution of the anorexigenic peptide leptin in the gastrointestinal tract of the bottlenose dolphin [36]. The physiological mechanisms that regulate glucose metabolism and food intake in cetaceans are not fully understood. Here we present a general approach to the presence and possible role of orexins in the gut of the bottlenose dolphin *Tursiops truncatus* (Montagu 1821): the expression and topography of the orexin system (OXA, OXB, OX1R, OX2R) in the gastrointestinal tract were investigated by single immunostaining; the expressions of prepro-orexin and orexin receptors were analyzed by western blotting analysis; and the co-localization of vasoactive intestinal peptide (VIP) and OX1R in the enteric nervous system (ENS) was examined by double immunostaining. Finally, peptide concentrations were measured in plasma samples collected at 10∶00 and 17∶30 hours to assess the possible diurnal variation of the plasma levels of OXA in this species.

## Materials and Methods

### Animals and tissue preparations

For the present study we used a series of samples of the gastrointestinal tract of three specimens of bottlenose dolphin *Tursiops truncatus* stored at the *Mediterranean marine mammal tissue bank* (MMMTB) of the Department of Comparative Biomedicine and Food Science of the University of Padova (http://www.marinemammals.eu). The MMMTB stores tissues from stranded animals or from marine mammals who died in captivity. The MMMTB is a recognized CITES institution (IT 020). The samples (see Table 1) were removed within a few hours after death. Each sample was divided into two portions. One portion was frozen and stored at −80°C, while the other was fixed in 10% buffered formalin and later embedded in paraffin. Sections were serially cut in 8 µm thick transversal sections and placed on slide glasses. Frozen plasma samples of two male bottlenose dolphin were obtained from the MMMTB of Padova. The MMMTB stores tissues from stranded animals or from marine mammals who died in captivity. The MMMTB is a recognized CITES institution (IT 020). Archival plasma samples stored in the Bank are excesses of blood sampled from captive dolphin living in different Aquariums or Sea-worlds during routine veterinary medical controls and sent to the MMMTB for laboratory controls and special analyses. No venipuncture was performed specifically for this study. Plasma samples were originally collected at 10∶00 am and 17∶30 pm in four different days. Each blood sample was drawn from the ventral surface of the flukes of trained dolphins, and collected into vacuum EDTA tubes. The plasma was then separated after centrifugation for 20 min at 2800 g (3500 rpm) and stored at 20°C.

**Table 1:** Specimens and samples used for immunohistrochemistry and western blotting analysis.

specimen	First chamber or forestomach	Second chamber or mainstomach	Third chamber or pyloric stomach	Proximalintestine	Distal intestine
ID 107 (♂)		**x**	**x**		
ID 110 (♂)	**x**	**x**	**x**	**x**	**x**
ID 139 (♂)	**x**	**x**	**x**	**x**	**x**

Also, we used as positive control archival samples of male Wistar rat duodenum (Harlan, Italy), stored in the Department of Veterinary Medicine and Animal Production of University of Naples Federico II. The samples were collected during a series of animal experiments for which approval was granted by the Italian Ministry of the University (now called “Ministry of Education”) according to the contemporary regulation on animal experimentation [37].

Use of archival samples is encouraged based on the EU Directive 2010/63/ of 22 September 2010 on the protection of animals used for scientific purposes: “Member States should, where appropriate, facilitate the establishment of programmes for sharing the organs and tissue of animals that are killed” (Introduction section #27).

### Single immunohistochemistry

The expression and distribution of OXA, OXB, OX1R and OX2R in the gastroenteric tract of the bottlenose dolphin were studied by immunohistochemistry. The sections were de-waxed and incubated with 0.3% hydrogen peroxide for 30 min at room temperature (RT), to block endogenous peroxidase activity. The sections were then rinsed in 0.01 M phosphate-buffered saline (PBS), pH 7.4, for 15 min and subsequently incubated for 20 min at RT with normal rabbit serum. Normal serum and the other components of the immunological reaction were contained in the Vectastain Elite ABC kit (PK 6105; Vector Laboratories Inc, CA, USA). In the specific step, polyclonal antibodies raised in goat against OXA (sc-8070), OXB (sc-8071), OX1R (sc-8072) and OX2R (sc-8074) from Santa Cruz Biotechnology Inc. (Santa Cruz, CA) were used and diluted 1∶300. After incubation with primary antisera the sections were rinsed in PBS for 15 min and incubated for 30 min at RT with biotinylated rabbit anti goat IgG. Subsequently, the sections were rinsed in PBS for 15 min and then incubated for 30 min at RT with an avidin-peroxidase complex. Peroxidase activity was detected using a solution of 3–3′ diaminobenzidine tetrahydrocloride (Sigma, St. Louis, MO, USA) of 10 mg in 15 ml of 0.5 M Tris buffer, pH 7.6, containing 0.03% hydrogen peroxide.

Antigen unmasking procedures always preceded the immunohistochemical reaction and were carried out by dipping the sections in 0.01 M sodium citrate buffer, pH 6.0, and heating them in a microwave oven for 10 min at 750 W.

### Double immunostaining

Double immunohistochemical staining was performed as follows: histological sections were de-waxed, rehydrated, rinsed in PBS and incubated for 30 min at RT with normal rabbit serum (1∶5; S-5000, Vector Laboratories Inc., Burlingame, CA). After blocking of endogenous biotin was performed (Avidin-Biotin blocking kit, SP-2001, Vector), the sections were incubated with the first primary antibody (goat anti-OX1R 1∶50) over night at 4°C. The sections were then washed with PBS and incubated with a biotinylated secondary rabbit-anti goat antibody (1∶20; BA-5000, Vector) for 2 h at RT. After rinsing in PBS, the sections were incubated with Streptavidin Texas Red conjugate (1∶50; S-872, Life Technologies Europe, Monza, Italy) for 2 h at RT in dark humid chamber. After rinsing in PBS the sections were incubated with normal donkey serum (1∶5; 017-000-121, Jackson Immunoresearch Laboratories Inc., Suffolk, UK) for 30 min at RT in dark humid chamber. The sections were subsequently incubated with the second primary antibody rabbit-anti VIP (1∶20; 20077, ImmunoStar, Inc., Hudson, WI, USA) over night at 4°C in dark humid chamber. After rinsing in PBS, the sections were incubated with secondary antibody Alexa Fluor 488-conjugated affinipure donkey anti-rabbit (1∶50, 711-545-152, Jackson) for 2 h at RT in dark humid chamber. Finally the sections were washed with PBS and mounted with glycerin diluted with PBS 1∶1.

Fluorescent and light images were observed and analyzed by Nikon Eclipse 90*i*. The digital raw images were optimized for image resolution, contrast, evenness of illumination, and background by using Adobe Photoshop CS5 (Adobe Systems, San Jose, CA).

### Controls

Controls of antibody specificity were performed by absorbing each primary antiserum with an excess of the relative peptide (100 mg of peptide/ml of diluted antiserum; OXA/sc-8070p; OXB/sc8071p; OX1R/sc-8072p; OX2R/sc-8074p; Santa Cruz). Internal reaction controls were carried out by substituting primary antisera or secondary antisera with PBS or normal serum in the specific step. Archival samples of rat duodenum, known to be positive to the orexin system, were used as positive reference controls.

### Western Blot Analysis

Bottlenose dolphin gastrointestinal tract and rat duodenum samples (1 g) were extracted in 6 ml of RIPA (Radio Immunoprecipitation Assay) buffer with Lysis buffer (50 mM Tris–HCl pH 7.4, 1% Triton X-100, 0.25% Na-deoxycholate, 150 mM NaCl, 1 mM EDTA), to which protein inhibitors 2 mM PMSF and protein inhibitor cocktail (P8340; Sigma-Aldrich) were added. Samples were homogenized with an Ultra-Turrax T25 (IKA-Labortechnik, Staufer, Germany) at 13,500 rpm. Homogenates were centrifugated 10,000 rpm for 20 min at 4°C, supernatants were collected separately and the protein concentration was determined with Bio-Rad dye protein assay (Bio-Rad Laboratories Inc., Hemel Hempstead, UK). 25 µg of protein for each sample were boiled at 98°C for 10 min in 25 µg of loading buffer 2x (50 mM Tris–HCl pH 6.8, 100 mM β-mercaptoethanol, 2% SDS, 0.1% blue bromophenol, 10% glycerol). Proteins were separated on a 12% (for orexins receptors) and 16% (for prepro-orexin) SDS–polyacrylamide gel electrophoresis with 4% stacking gel in 1% Tris–glycine buffer (0.025 M Tris, 0.192 M glycine, and 0.1% SDS [pH 8.3]) in a miniprotean cell (Bio-Rad) at 130 V for 2 h. The separated proteins were electrotransferred onto a polyvinylidene fluoride membrane with transfer buffer (25 mM Tris base, 0.2 M glycine, and 20% methanol [pH 8.5]) in a minitransfer cell (Bio-Rad) at 100 V at 4°C for 1 h. Membranes were incubated at 4°C for 1 h in blocking buffer containing 1% PBS and 0.05% Tween 20 with 5% dried non-fat milk and then were probed with polyclonal antibodies in rabbit raised against prepro-orexin AB3096, Millipore, Temecula, CA, USA) and β-actin (A5060, Sigma, Sant Louis, MO, USA), used as internal marker, and polyclonal antibodies in goat raised against OX1R (sc-8072, Santa Cruz Biotechnologies Inc) and OX2R (sc-8074, Santa Cruz Biotechnologies Inc.). All primary antibodies were diluted 1∶1,000 and incubated over night at 4°C. This was followed by incubation with the secondary rabbit anti-goat IgG (Millipore 1∶5000), and goat anti-rabbit IgG (Sigma 1∶5000) antibodies for 1 h at RT. Signals were detected by chemoluminescence with the Pico Enhanced Chemiluminescence Kit (Pierce Chemical) with Chemidoc (Bio-Rad). A pre-stained molecular-weight ladder (Novex Sharp Pre-Stained Protein Standard, Life Technologies, Monza, Italy) was used to determine protein size.

Specificity was determined by pre-absorption of primary antibodies with their relative control peptides before western blotting.

### Orexin A Plasma Levels

Plasma OXA concentration was measured with an enzyme immunoassay kit for orexin A/Hypocretin-1 (EK-003-30; Phoenix Pharmaceuticals Inc, Burlingame, CA, USA), according to the manufacturer’s protocols. The assay was validated with a dilution series. The standard curve was 0.01–1,000 ng/mL. According to the manufacturer, OXA antibody cross-reacted with human, rat, mouse and bovine orexin A. The absorbance was read at 450 nm with a microplate reader (Bio-Rad, model 680).

### Statistical Analysis

Data were analyzed by one-way analysis of variance (ANOVA) and any significant difference was determined at a significance level of 0.05 via the application of a Tukey’s test. The analyses were carried out using Statistica version 7.0 (Statsoft inc., Tulsa, OK, USA).

## Results

The post-diaphragmatic gastrointestinal tract of the bottlenose dolphin includes a three chambered stomach, a pyloric stomach and a long undivided, unvarying intestine. Since a division between small and large intestine (and respective sub-constituent parts) is impossible to perform, here we considered a proximal and a distal part of the intestine based on their approximate length.

### Single Immunohistochemistry

**Figure 1:**
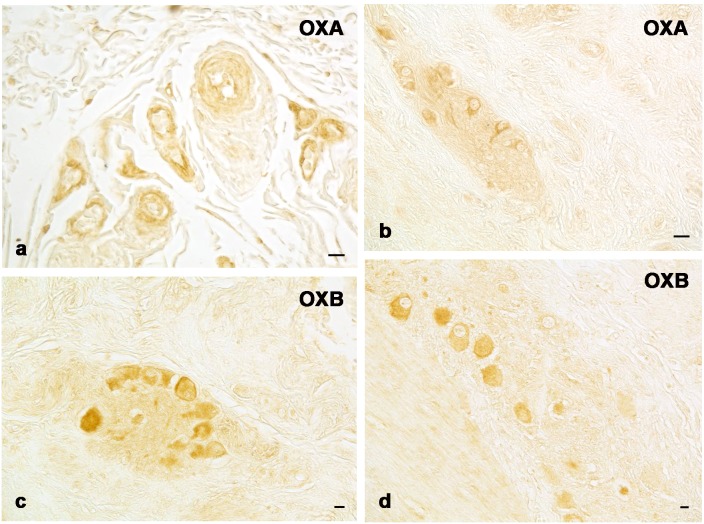
OXA and OXB single immunohistochemical detection. OXA immunopositive perivascular fibers in the submucosal layer of main stomach (a); OXA ir neurons and nervous fibers in the myenteric plexus of proximal intestine (b); OXB ir neurons of the submucosal plexus of pyloric stomach (c) and myenteric plexus of proximal intestine (d). Scale bars: 5 µm (a); 20 µm (b); 10 µm (c, d).

Orexin system immunopositivity was widely distributed throughout the entire bottlenose dolphin gastrointestinal tract. In particular, immunopositivity was evident in the ENS. OXA immunoreactive (ir) perivascular (Fig. 1a), and ganglion nervous fibers, as well as neuronal cells (Fig. 1b) were detected both in the submucosal layer (Fig. 1a) and the myenteric plexuses of the main stomach, pyloric stomach and proximal intestine (Fig. 1b). OXB-ir (Fig. 1c, d) neurons and fibers were found in the ENS of the same gastrointestinal tract.

**Figure 2:**
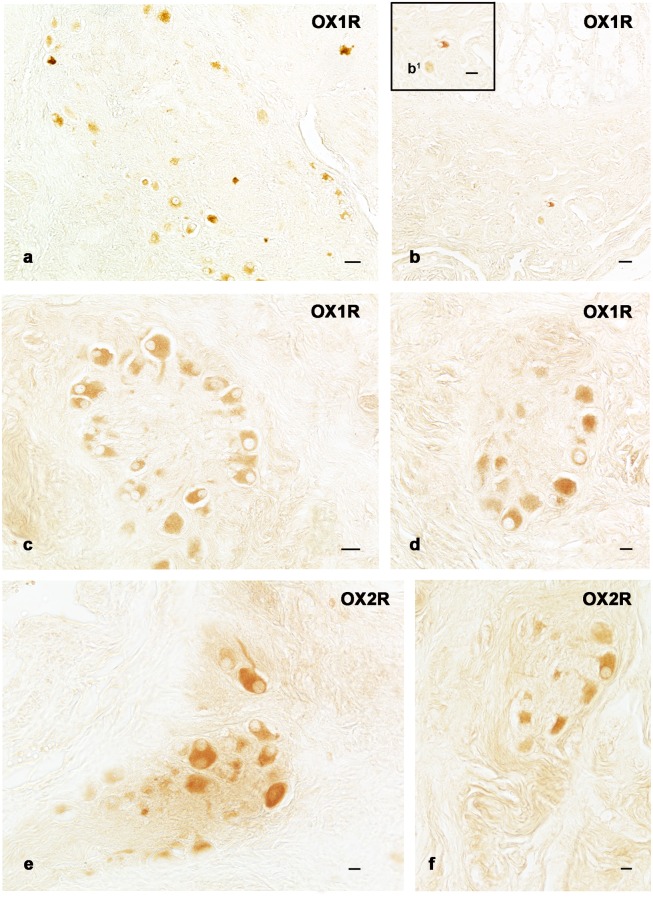
OX1R and OX2R single immunohistochemical detection. Orexin 1 (a–d) and orexin 2 (e, f) receptors ir nervous fibers and neurons in the submucosal plexus of forestomach (a), proximal (b, b^1^; b^1^ detail of b) and distal (e) intestine and in the myenteric plexus of proximal (c) and distal (d, f) intestine. Scale bars: 30 µm (a, b); 20 µm (c); 10 µm (d–f).

OX1R-ir neurons and fibers were identified in both plexuses of the ENS (Fig. 2 a–d) throughout the gut. Immunostained neurons were occasionally found scattered (Fig. 2a) or isolated (Fig. 2b, b ^1^), but in most cases they were grouped and belonged to the ganglion structures (Fig. 2c, d). OX2R-ir neurons and fibers were observed in the nervous plexuses of the intestinal tracts (Fig. 2e, f). A high intensity of staining was detected in both the submucosal (Fig. 2e) and myenteric ganglion (Fig. 2f) of the distal intestine.

### Double Immunohistochemistry

**Figure 3:**
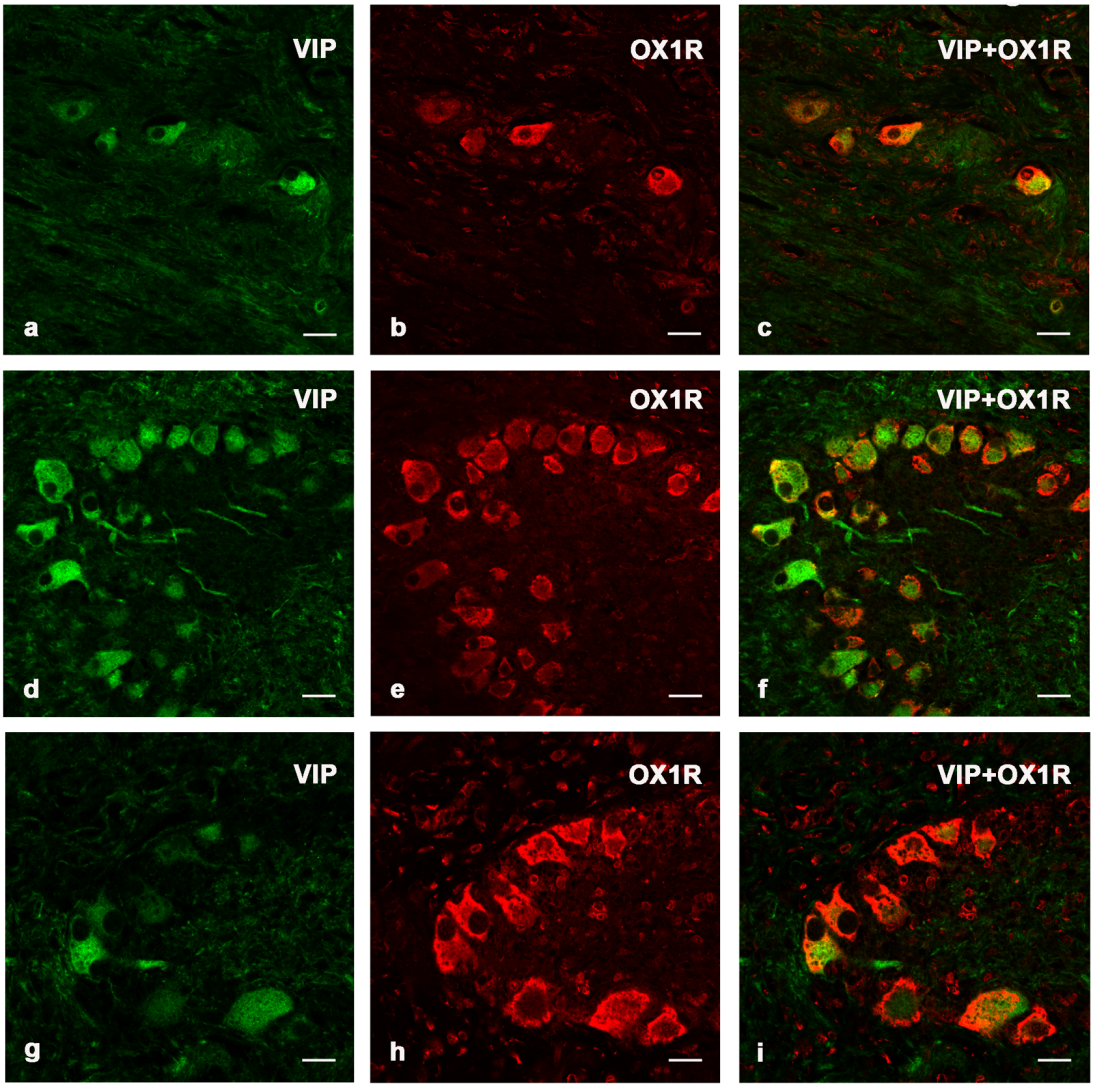
VIP and OX1R double immunostaining. Colocalizations of VIP- (green) and OX1R- (red) immunopositive neurons and nervous fibers located in the myenteric plexus of pyloric stomach (a–c) and submucosal plexus of proximal (d–f) and distal (g–i) intestine. Scale bars: 20 µm (a–i).

Double immunostaining allowed the identification of a population of neurons and fibers that were positive to both VIP and OX1R. The immunopositive nervous elements were found both in submucosal and in the myenteric plexuses of the pyloric stomach (Fig. 3a–c), proximal (Fig. 3d–f) and distal intestine (Fig. 3g–i). Co-localizations of VIP-ir and OX1R-ir elements were more evident in the pyloric stomach and proximal intestine than in the distal intestine.

### Protein expression

**Figure 4:**
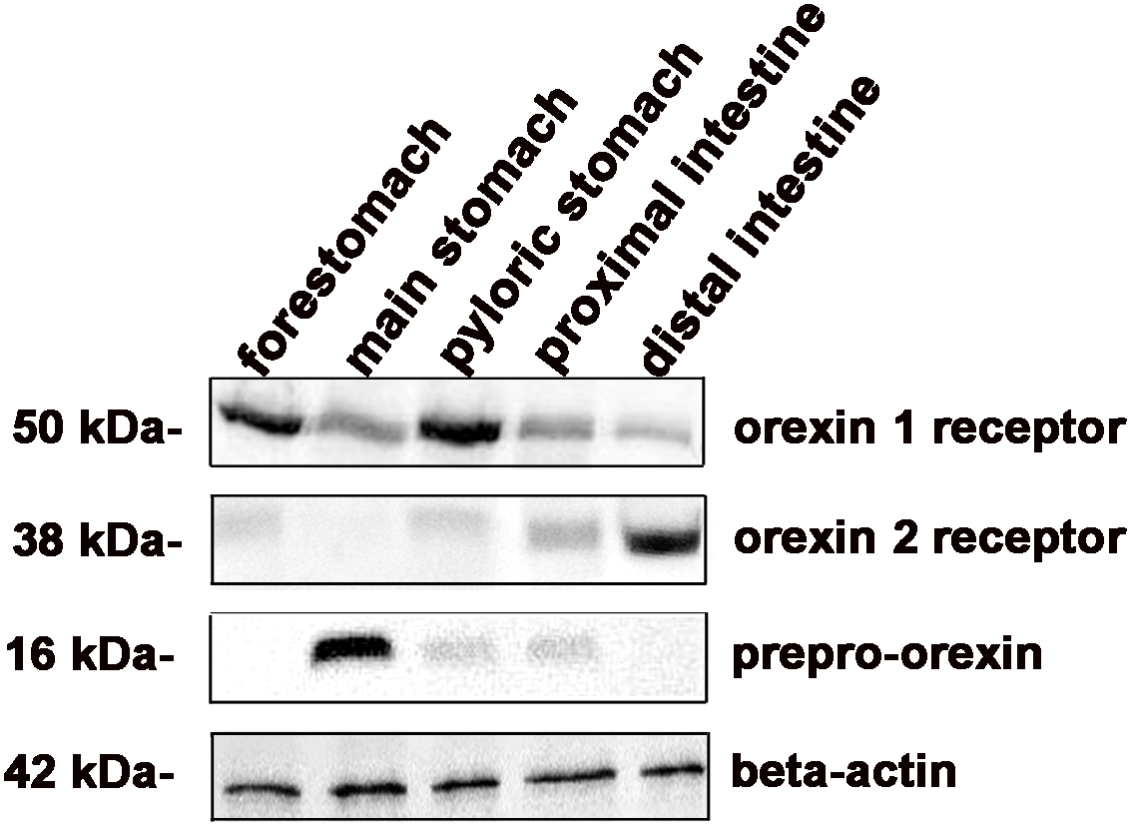
Orexin system protein expression. Western blot for orexin 1 and 2 receptors, prepro-orexin and β-actin (used as internal marker) in forestomach, main stomach, pyloric stomach, proximal and distal intestine.

Western blot analysis was carried out on homogenates of all gastrointestinal tract of bottlenose dolphin (forestomach, main stomach, pyloric stomach, proximal and distal intestine). The results of western blot analysis (Fig. 4) showed the presence of prepro-orexin 16 kDa band in the main stomach, pyloric stomach and proximal intestine. The presence of OX1R immunoreactive band of about 50 kDa and OX2R immunoreactive band of about 38 kDa was detected in all gastrointestinal tracts, with the exception of main stomach where no band for OX2R was present. β-actin, used as internal marker, was detected in all examined samples as a band of about 42 kDa.

The specificity of the response was confirmed by pre-incubation of prepro-orexin, OX1R and OX2R antibodies with their respective blocking peptides. There was no expression of prepro-orexin, Ox1R and OX2R in these preparations, whereas the presence of the proteins was detected in rat duodenum homogenate that was used as positive control.

### Orexin A Plasma Levels

**Figure 5:**
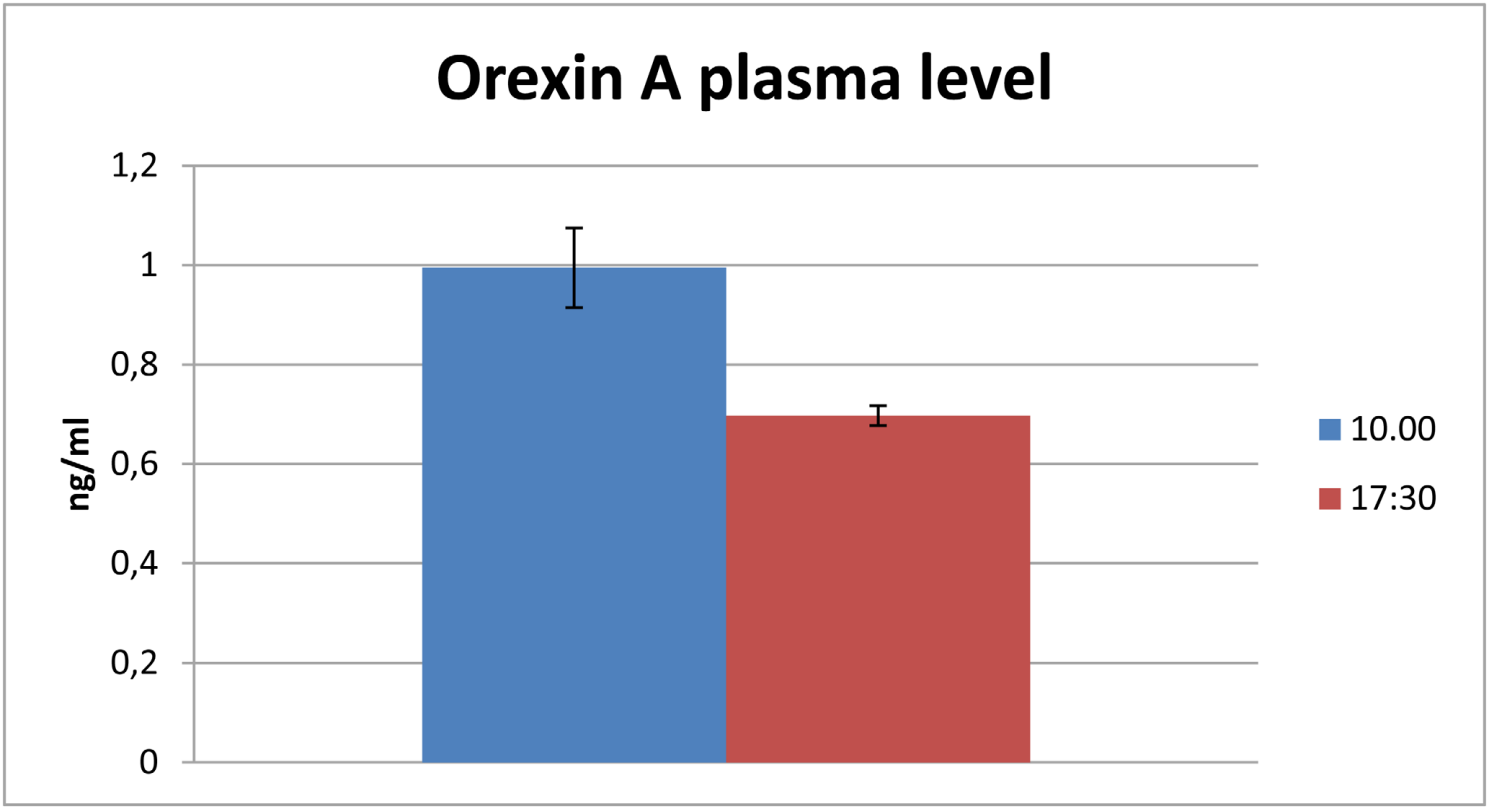
OXA plasma levels. Bottlenose dolphin plasma OXA concentration (mean ± SD) at 10∶00 and 17∶30 hours.

OXA plasma concentrations are reported in Figure 5. OXA levels were statistically higher in the morning (10∶00) than in the afternoon (17∶00).

## Discussion

In the last decade, numerous investigations report the presence and the expression of the orexin system in the gastrointestinal tract of several vertebrates [22], [23], [38], [39]. The distribution of orexins and their specific receptors along the gastrointestinal tract varies in terrestrial mammals, depending on the species of animal investigated [40]–[42] and the respective digestive physiology. Toothed whales, including the bottlenose dolphin, are entirely carnivore species that feed mainly on fish or marine invertebrates. Dolphins do not chew their food, but grab and swallow their prey whole, leaving mechanical digestion to the heavily muscular forestomach [43]. The intestine shows no macroscopic or microscopic subdivisions into a small or large part, but consists of a long unvarying tube of homogenous caliber. Our results showed that in gastrointestinal tract of the bottlenose dolphin the distribution of the orexin system is very peculiar and partially differs from that of terrestrial mammals. OXA, OXB and prepro-orexin are expressed in the main stomach, pyloric stomach and proximal intestine. Orexin receptors are expressed in all the examined tracts, with the exception of main stomach where we found no evidence of OX2R. Therefore our data suggest that the orexin system is widely distributed in bottlenose dolphin ENS. The majority of neurons and fibers with high intensity of staining were identified in the intestinal tract, as also observed in newborn dogs and pigs (carnivorous and omnivorous monogastric animals) [40], [42] and in fallow deer (erbivorous poligastric mammal) [44]. The abundant expression of orexins in the post-diaphragmatic gastrointestinal tract of the bottlenose dolphin supports the involvement of orexigenic peptides in the regulation of gastrointestinal secretion and motility, as suggested for terrestrial mammals, in which orexin peptides modulate digestive physiology acting centrally or peripherally. The specific anatomy of the intestine of dolphins may explain the apparent uniform distribution of both orexin and their respective receptors.

OXA penetrates the blood-barrier by simple diffusion, contrarily to OXB [45], and this may explain why the different effects of the two peptides. OXA stimulates gastric acid secretion throughout vagal pathways [46], regulates intestinal bicarbonate secretion [32], and gastrointestinal and colon motility [33], [35], [47]. Studies on the dose-dependent peripheral effects of OXA and OXB on intestinal wall show that they slow down intestinal motility by extending of duration of migrating motor complex (MMC) [22,23,48 7].

Our double immunostaining results, in agreement with Naslünd et al. (2002), showed OX1R and VIP-ir neurons and fibers in submucosal and myenteric plexuses of the pyloric stomach, proximal and distal intestine. Co-localizations of VIP and OX1R were more evident in the pyloric stomach and proximal intestine than in the distal intestine. VIP exerts various effects on the gastrointestinal tract of terrestrial mammals, including the inhibition of MMC [49]. VIP acts on MMC directly, or indirectly either through l-arginine/nitric oxide, or OXA/OX1R pathways [22], [50]. Its co-localization with OX1R could suggest that orexin system act on the intestinal motility also in the bottlenose dolphin, reducing the contractility in the gastrointestinal districts similarly to what described in terrestrial mammals [33], [51].

Gastrointestinal and blood samples used in this study were collected during routine veterinary medical procedures from captive animals used to a constant feeding schedule including regular meals and feeding sessions used to positively enforce required behaviors. Since blood cannot be drawn from wild animals, possible differences with free-ranging individuals should be taken into account. However bottlenose dolphins do not usually undergo long migrations to reach breeding grounds and generally show no fasting period during the mating season or calving. However our data indicate that plasma concentrations of OXA in bottlenose dolphins are higher in the morning before their main meal. This is in agreement with several studies reporting the increase of plasma OXA concentration during fasting in rats and human [22], [52], [53]. A correlation was found in the bottlenose dolphin between urinary vasopressin values after the meal and urine concentration, a sign that indicates conservation of food-derived water [54]. Orexin concentration may have a direct relationship with the glucose requirements of the brain, and the specific needs of the peculiar central blood flow and metabolism typical of dolphins [55]. However the presence and nature of circadian and circannual rhythms in dolphins remain open, at least based on the origin and concentration of neuroendocrine transducer melatonin [56]. Furthermore, the neurophysiology of sleep and the alternate phases of rest-activity cycles are markedly different in cetaceans [57].

Physiological investigations on marine mammals are obviously mostly limited to observation of wild specimens and occasional studies on captive animals. Feeding strategies, food preferences and even diving behavior can be studied in wild populations of bottlenose dolphins, but actual information on digestive physiology derive largely from trained dolphins living within a confined environment. So i.e. the peculiar glucose metabolism of the bottlenose dolphin that shows postprandial hyperglycemia similar to humans with diabetes mellitus type two [58], [59] remains largely unexplained. However orexin concentration may have a direct relationship with - or be linked to - the glucose requirements of the brain, and thus also be part of the mechanism of selective control of central blood flow and metabolism, important for the integration of auditory and other sensory inputs [55]. We also note that unihemispheric sleep implies uneven distribution of glucose between the active and resting parts of the brain [60].

Our report adds novel information on the presence and distribution of the orexin system in the gastrointestinal tract of dolphins. These results, taken together with data on the plasma concentration of orexins, emphasize some common features between bottlenose dolphin and terrestrial mammals. Further functional investigations may help to better explain the role of the orexin system in the energy balance of bottlenose dolphin and the complex interaction between feeding and digestive physiology.

## References

[1] Reidenberg J (2007) Anatomical adaptations of aquatic mammals. Anat Rec 290: 507–513. 10.1002/ar.20541 17516440

[2] Guimarães JP, Mari RB, Marigo J, Rosas FCW, Watanabe I (2012) Gross and microscopic observations on the lingual structure of the Franciscana (*Pontoporia blainvillei -* Gervais and d’Orbigny, 1844). Microscopy Res Techn 75: 737–742. 10.1002/jemt.21119 22298326

[3] Barros NB, Clarke MR (2008) Diet. In: Encyclopedia of marine mammals, II edition (Perrin WF, Würsing B, and Thewissen JGM, editors), Academic Press, Amsterdam, 311–316.

[4] Worthy GAJ (2001) Nutrition and energetics. In: CRC Handbook of marine mammal medicine, II edition (Dierauf LA and Gulland FMD, editors), CRC Press, Boca Raton, 791–827.

[5] Sakurai T, Amameya A, Ishii M, Matsuzaki I, Chemelli RM, et al (1998) Orexins and orexin receptors: a family of hypothalamic neuropeptides and G protein-coupled receptors that regulate feeding behaviour. Cell 92: 573–585. 9491897 10.1016/s0092-8674(00)80949-6

[6] de Lecea L, Kilduff TS, Peyron C, Gao X-B, Foye PE, et al (1998) The hypocretins: hypothalamus-specific peptides with neuroexcitatory activity. Proc Nat Acad Sci 95: 322–327. 9419374 10.1073/pnas.95.1.322 PMC18213

[7] Kukkonen JP, Holmqvist T, Ammoun S, Akerman KEO (2002) Functions of the orexinergic/hypocretinergic system. Am J Physiol Cell Physiol 283: 1567–1591. 10.1152/ajpcell.00055.2002 12419707

[8] Yokobori E, Kojima K, Azuma M, Kang KS, Maejima S, et al (2011) Stimulatory effect of intracerebroventricular administration of orexin A on food intake in the zebrafish, *Danio rerio* . Peptides 32: 1357–1362. 21616109 10.1016/j.peptides.2011.05.010

[9] Facciolo MF, Crudo M, Zizza M, Giusi G, Canonaco M (2011) Feeding behaviors and ORXR-β-GABAAR subunit interactions in *Carassius auratus* . Neurotox and Teratol 33(6): 641–650. 10.1016/j.ntt.2011.09.008 22001787

[10] Nambu T, Sakurai T, Mizukami K, Hosoya Y, Yanagisawa M, et al (1999) Distribution of orexin neurons in the adult rat brain. Brain Res 827: 243–260. 10320718 10.1016/s0006-8993(99)01336-0

[11] Sakurai T (2006) Roles of orexins and orexin receptors in the central regulation of feeding behavior and energy homeostasis. CNS Neurol Disord Drug Targets 5: 131–325. 10.2174/187152706777452218 16787231

[12] Matsuda K, Azuma M, Kang KS (2012) Orexin system in teleost fish. Vitam Horm 89: 341–346. 22640622 10.1016/B978-0-12-394623-2.00018-4

[13] Miranda B, Esposito V, de Girolamo P, Sharp PJ, Wilson PW, et al (2013) Orexin in the chicken hypothalamus: immunocytochemical localisation and comparison of mRNA concentrations during the day and night, after chronic food restriction. Brain Res 1513: 34–40. 23548597 10.1016/j.brainres.2013.03.036

[14] Nakabayashi M, Suzuki T, Takahashi K, Totsune K, Muramatsu Y, et al (2003) Orexin-A expression in human peripheral tissue. Mol Cell Endocrinol 205: 43–50. 12890566 10.1016/s0303-7207(03)00206-5

[15] Digby JE, Chen J, Tang JY, Lehnert H, Matthews RN, et al (2006) Orexin receptor expression in human adipose tissue: effects of orexin-A and orexin-B. J Endocrinol 191: 129–136. 17065396 10.1677/joe.1.06886

[16] Takahashi K, Arihara Z, Suzuki T, Sone M, Kikuchi K, et al (2006) Expression of orexin-A and orexin receptors in the kidney and the presence of orexin-A-like immunoreactivity in human urine. Peptides 27: 871–877. 16202475 10.1016/j.peptides.2005.08.008

[17] Kirchgessner AL, Liu M (1999) Orexin synthesis and response in the gut. Neuron 24: 941–951. 10624957 10.1016/s0896-6273(00)81041-7

[18] Randeva HS, Karteris E, Grammatopoulos D, Hillhouse EW (2001) Expression of orexin-A and functional orexin type 2 receptors in human adult adrenals: implications for adrenal function and energy homeostasis. J Clin Endocrinol Metab 86: 4808–4813. 11600545 10.1210/jcem.86.10.7921

[19] Johren O, Neidert SJ, Kummer M, Dendorfer A, Dominiak P (2001) Prepro-orexin and orexin receptor mRNAs are differentially expressed in peripheral tissues of male and female rats. Endocrinology 142: 3324–3331. 11459774 10.1210/endo.142.8.8299

[20] Russo F, Pavone LM, Tafuri S, Avallone L, Staiano N, et al (2008) Expression of orexin A and its receptor 1 in the bovine urethroprostatic complex. Anat Rec 291: 169–174. 10.1002/ar.20641 18213704

[21] Russo F, Maruccio L, Calamo A, de Girolamo P, Varricchio E (2014) Orexin 1 receptor in the seminiferous tubules of boar testis: immunohistochemical study. Acta Histochem 116: 286–8. 23746541 10.1016/j.acthis.2013.04.012

[22] Näslund E, Ehström M, Ma J, Hellström PM, Kirchgessner AL (2002) Localization and effects of orexin on fasting motility in the rat duodenum. Am J Physiol Gatrointest Liver Physiol 282: G470–G479. 10.1152/ajpgi.00219.2001 11841997

[23] Ehström M, Gustafsson T, Finn A, Kirchgessner A, Gryback P, et al (2005) Inhibitory effect of exogenous orexin A on gastric emptying, plasma leptin, and the distribution of orexin and orexin receptors in the gut and pancreas in man. J Clin Endocrinol Metab 90: 2370–2377. 15671114 10.1210/jc.2004-1408

[24] Shirasaka T, Nakazato M, Matsukura S, Takasaki M, Kannan H (1999) Sympathetic and cardiovascular actions of orexins in conscious rats. Am J Physiol 277: R1780–R1785. 10600926 10.1152/ajpregu.1999.277.6.R1780

[25] Piper DC, Upton N, Smith MI, Hunter AJ (2000) The novel brain neuropeptide, orexin-A, modulates the sleep-wake cycle in rats. Eur J Neurosci 12: 726–730. 10712652 10.1046/j.1460-9568.2000.00919.x

[26] Kunii K, Yamanaka A, Nambu T, Matsuzaki I, Goto K, et al (1999) Orexins/hypocretins regulate drinking behavior. Brain Res 842: 256–261. 10526122 10.1016/s0006-8993(99)01884-3

[27] Kuru M, Ueta Y, Serino R, Nakazato M, Yamamoto Y, et al (2000) Centrally administered orexin/hypocretin activates HPA axis in rats. Neuroreport 11: 1977–1980. 10884055 10.1097/00001756-200006260-00034

[28] Barreiro ML, Pineda R, Gaytan F, Archanco MA, Burrell MA, et al (2005) Pattern of orexin expression and direct biological actions of orexin-A in rat testis. Endocrinology 146: 5164–5175. 16141395 10.1210/en.2005-0455

[29] Ouedraogo R, Naslünd E, Kirchgessner AL (2003) Glucose regulates the release of orexin-a from the endocrine pancreas. Diabetes 52: 111–117. 12502500 10.2337/diabetes.52.1.111

[30] Nowak KW, Strowski MZ, Switonska MM, Kaczmarek P, Singh V, et al (2005) Evidence that orexins A and B stimulate insulin secretion from rat pancreatic islets via both receptor subtypes. Int J Mol Med 15: 969–972. 15870901

[31] Miyasaka K, Masuda M, Kanai S, Sato N, Kurosawa M (2002) Central orexin-A stimulates pancreatic exocrine secretion via the vagus. Pancreas 25: 400–404. 12409836 10.1097/00006676-200211000-00013

[32] Flemström G, Bengtsson MW, Mäkela K, Herzig H (2010) Effects of short-term food deprivation on orexin-A-induced intestinal bicarbonate secretion in comparison with related secretagogues. Acta Physiol 198: 373–380. 10.1111/j.1748-1716.2009.02067.x 20003099

[33] Bülbül M, Babygirija R, Zheng J, Ludwig KA, Takahashi T (2010a) Central orexin-A changes the gastrointestinal motor pattern from interdigestive to postprandial in rats. Auton Neurosci 158: 24–30. 20542473 10.1016/j.autneu.2010.05.009

[34] Chaleek N, Kermani M, Eliassi A, Haghparast A (2011) Effects of orexin and glucose microinjected into the hypothalamic paraventricular nucleus on gastric acid secretion in conscious rats. Neurogastroenterol Motil 24: 94–102. 10.1111/j.1365-2982.2011.01789.x 22004243

[35] Nozu T, Kumei S, Takakusaki K, Ataka K, Fujimiya M, et al (2011) Central orexin-A increases colonic motility in conscious rats. Neurosci Lett 498: 143–146. 21575675 10.1016/j.neulet.2011.04.078

[36] Russo F, Gatta C, de Girolamo P, Cozzi B, Giurisato M, et al (2012) Expression and immunohistochemical detection of leptin-like peptide in the gastropintestinal tract of the South American Sea Lion (*Otaria flavescens*) and bottlenose dolphin (*Tursiops truncatus*). Anat Rec 295: 1482–1493. 10.1002/ar.22532 22791650

[37] Tafuri S, Pavone LM, Lo Muto R, Basile M, Langella E, et al (2009) Expression of orexin A and its receptor 1 in the rat testis. Regul Pept 155: 1–5. 19328827 10.1016/j.regpep.2009.03.010

[38] Kirchgessner LA (2002) Orexins in the brain-gut axis. Endocr Rev 23: 1–15. 11844742 10.1210/edrv.23.1.0454

[39] Mac Donald EE, Volkoff H (2010) Molecular cloning and characterization of preproorexin in winter skate (*Leucoraja ocellata*). Gen Comp Endocrinol 169: 192–196. 20875823 10.1016/j.ygcen.2010.09.014

[40] Dall’Aglio C, Pascucci L, Mercati F, Giontella A, Pedini V, et al (2008) Identification of orexin a and orexin type 2 receptor-positive cells in the gastrointestinal tract of neonatal dogs. Eur J Histochem 52: 229–235. 19109097 10.4081/1221

[41] Dall’Aglio C, Pascucci L, Mercati F, Giontella A, Pedini V, et al (2009) Immunohistochemical identification and localization of orexin A and orexin type 2 receptor in the horse gastrointestinal tract. Res Vet Sci 86: 189–193. 18707745 10.1016/j.rvsc.2008.07.001

[42] Dall’Aglio C, Zannoni A, Forni M, Bacci ML, Ceccarelli P, et al (2013) Orexin system expression in the gastrointestinal tract of pig. Res Vet Sci 95: 8–14. 23485172 10.1016/j.rvsc.2013.02.001

[43] Harrison RJ, Johnson FR, Young BA (1970) The oesophagus and stomach of dolphins (*Tursiops*, *Delphinus*, *Stenella*). J Zool 160: 377–390.

[44] Dall’Aglio C, Pascucci L, Mercati F, Boiti C, Ceccarelli P (2012) Localization of the orexin system in the gastrointestinmal tract of fallow deer. Acta Histochem 114: 74–78. 21397934 10.1016/j.acthis.2011.02.006

[45] Kastin AJ, Åkerström V (1999) Orexin A but not orexin B rapidly enters brain from blood by simple diffusion. J Pharmacol Exp Ther 289: 219–223. 10087007

[46] Okumura T, Takakusaki K (2008) Role of orexin in central regulation of gastrointestinal functions. J Gastroenterol 43: 652–660. 18807126 10.1007/s00535-008-2218-1

[47] Kobashi M, Furudono Y, Matsuo R, Yamamoto T (2002) Central orexin facilities gastric relaxation and contractility in rats. Neurosci Lett 332: 171–174. 12399008 10.1016/s0304-3940(02)00958-8

[48] Bülbül M, Tan R, Gemici B, Özdem S, Üstünel I, et al (2010b) Endogenous orexin-A modulates gastric motility by peripheral mechanisms in rats. Peptides 31: 1099–1108. 20307611 10.1016/j.peptides.2010.03.007

[49] Ljung T, Hellström PM (1999) Vasoactive intestinal peptide suppresses migrating myoelectric complex of rat small intestine independent of nitric oxide. Acta Physiol Scand 165: 225–231. 10090335 10.1046/j.1365-201x.1999.00497.x

[50] Hellström PM, Ljiung T (1996) Nitrergic inhibition of migrating myoelectric complex in the rat is mediated by vasoactive intestinal peptide. Neurogastroenterol Motil 8: 299–306. 8959734 10.1111/j.1365-2982.1996.tb00268.x

[51] Nakayama S (2011) Orexins stimulate the “appetite” of the gut. J Physiol 589: 5907–5908. 22174137 10.1113/jphysiol.2011.220962 PMC3286666

[52] Korczynski W, Ceregrzyn M, Matyek R, Kato I, Kuwahara A, et al (2006) Central and local (enteric) action of orexins. J Physiol Pharmacol 57: 17–42. 17228085

[53] Komaki G, Matsimoto Y, Nishikata H, Kawai K, Nozaki T, et al (2001) Orexin-A and leptin change inversely in fasting non-obese subjects. Eur J Endocrinol 144: 645–651. 11375799 10.1530/eje.0.1440645

[54] Ballarin C, Corain L, Peruffo A, Cozzi B (2011) Correlation between urinary vasopressin and water content of food in the bottlenose dolphin (*Tursiops truncatus*). Open Neuroendocr J 4: 9–14.

[55] Houser DS, Moore PW, Johnson S, Lutmerding B, Branstetter B, et al (2010) Relationship of blood flow and metabolism to acoustic processing centers of the dolphin brain. J Acoust Soc Am. 128: 1460–1466. 10.1121/1.3442572 20815480

[56] Panin M, Gabai G, Ballarin C, Peruffo A, Cozzi B (2012) Evidence of melatonin secretion in cetaceans: plasma concentration and extrapineal HIOMT-like presence in the bottlenose dolphin *Tursiops truncatus*. Gen Comp Endocrinol. 177: 238–245. 10.1016/j.ygcen.2012.04.012 22554922

[57] Lyamin OI, Manger PR, Ridgway SH, Mukhametov LM, Siegel JM (2008) Cetacean sleep: an unusual form of mammalian sleep. Neurosci Biobehav Rev 32: 1451–1484. 18602158 10.1016/j.neubiorev.2008.05.023 PMC8742503

[58] Venn-Watson S, Carlin K, Ridgway S (2011) Dolphins as animal models for type 2 diabetes: sustained, post-prandial hyperglycemia and hyperinsulinemia. Gen Comp Endocrinol 170: 193–199. 20951701 10.1016/j.ygcen.2010.10.005

[59] Venn-Watson S, Rowe Smith C, Stevenson S, Parry C, Daniels R, et al (2013) Blood-based indicators of insulin resistance and metabolic syndrome in bottlenose dolphins (*Tursiops truncatus*). Front Endocrinol 4: 136. 10.3389/fendo.2013.00136 PMC3793200 24130551

[60] Ridgway S, Houser D, Finneran J, Carder D, Keogh M, et al (2006) Functional imaging of dolphin brain metabolism and blood flow. J Exp Biol. 209: 2902–2910. 10.1242/jeb.02348 16857874

